# Breeder turnover creates allelic variation in groups of gray wolves

**DOI:** 10.1038/s41437-025-00788-4

**Published:** 2025-09-23

**Authors:** David E. Ausband

**Affiliations:** https://ror.org/03hbp5t65 grid.266456.5 0000 0001 2284 9900 U.S. Geological Survey, Idaho Cooperative Fish and Wildlife Research Unit, University of Idaho, Moscow, ID USA

**Keywords:** Ecological genetics, Behavioural ecology

## Abstract

Genetic diversity is an important driver affecting the health of wildlife populations. In cooperatively breeding species, human impacts and breeder turnover can affect genetic diversity in groups. We generally do not have strong inferences about how the genetic composition of a group changes through time as individuals are lost (e.g., die, emigrate) or adopted (e.g., immigrate). I wanted to know how breeder turnover, group size, and harvest affected the fluctuation of unique alleles in groups of gray wolves (*Canis lupus*) in Idaho, USA, during 2008–2020. Turnover of breeding males was strongly associated with allelic change in groups. Turnover of breeding females also had a strong association with allelic change in groups, but was not the most supported model. Harvest was strongly correlated with breeding female turnover but not breeding male turnover. Outside of breeding female turnover, harvest generally had little effect on allelic change in groups. Groups rarely adopted new individuals unless there was a breeding vacancy. I show that over time groups gain and lose alleles in roughly equal proportions, but there are episodic changes to alleles in groups as a function of breeding male turnover. These findings have implications for how we define and evaluate group persistence and breeder lineages in cooperative breeders. Such definitions have important implications for studying the evolution and maintenance of cooperative breeding. It may be beneficial to define characteristics and vital rates of groups based, at least in part, on their underlying genetics when such information can be obtained.

## Introduction

Genetic diversity is an important driver affecting the health of wildlife populations (Allendorf et al. 2022). Genetic diversity can be affected by many factors including a species’ mating system, whether they are r or K-selected, as well as a host of ecological factors such as population size and the ability for animals to move and successfully intermix and breed (Mills 2013; Allendorf et al. 2022). Wolverines (*Gulo gulo*), for example, tend to have relatively low genetic diversity (Ekblom et al. 2018), in part, due to a polygamous mating system and relatively low population densities. Genetic diversity can also vary due to a species’ evolutionary history (e.g., Brown 1971; Ekblom et al. 2018).

Some animal populations have become less genetically diverse due to human impacts on their populations (Allendorf et al. 2022). Habitat alteration from human land use, for example, can cause habitat fragmentation and reduce genetic diversity in wild populations (Schlaepfer et al. 2018). Animals that live and breed in groups (i.e., cooperative breeders; Solomon and French 1997) could conceivably be more affected by human impacts due to limited breeding opportunities in their populations and low effective population sizes relative to other species that mate randomly.

For cooperative breeders, breeder turnover and mortality can greatly affect genetic diversity in the group (Ross 2001; Ausband et al. 2025). Group size might also affect genetic diversity because smaller groups may be more likely to experience genetic changes when there is breeder turnover or losses of individuals. Human impacts on the genetic diversity of groups of cooperative breeders can sometimes be marked. For example, poaching in a population of African elephants (*Loxodonta africana*) changed the social structure of family groups and reduced genetic relatedness to near 0.0 in highly disrupted groups (Gobush et al. 2009). Losses of genetic diversity and even increased hybridization due to human-caused mortality have been observed across several species of cooperative breeders such as gray wolves (*Canis lupus*), African elephants, and red wolves, (*Canis rufus*, Ausband et al. 2025). By contrast, relatively low levels of human harvest could conceivably increase genetic diversity in a population of cooperative breeders by creating breeding vacancies and facilitating gene flow among groups provided such dispersing individuals are not disproportionately harvested (Ausband et al. 2025)

While humans can affect the genetic diversity in a group of cooperative breeders, such groups can quickly gain back lost diversity by adopting unrelated individuals into the group (i.e., immigration) or even through multiple breeding (Rubenstein 2007; Chen et al. 2019). For cooperative breeders that commonly live in family groups comprising parents and several generations of offspring, one might expect the genetic composition of such groups to be fairly stable over time (i.e., most individuals’ genotypes reflect the parents). Groups in the wild are seldom unchanging, however, and we generally do not have strong inferences about how the genetic composition of a group changes through time as individuals are lost (e.g., die, emigrate) or adopted (e.g., immigrate).

Gray wolves in Idaho, USA, are an ideal species for asking questions about how human-caused mortality might affect the genetic composition of groups of cooperative breeders. Wolves are territorial carnivores that live in family groups (Boyd et al. 2023) and although a territory may be continually occupied over time, the individuals in that territory can fluctuate greatly as individuals are lost or adopted into the group (e.g. group augmentation). Reproduction is typically dominated by a breeding pair where 2–3 generations of offspring from prior years help rear the current year’s pups. Nonbreeding male and female wolves can disperse from their natal group and attempt to either establish their own group and territory elsewhere or join an existing group if possible. Wolves are hunted and trapped annually in Idaho and a large number of wolf groups experience at least some human-caused mortality each year providing an excellent opportunity ask questions about how the genetic composition of a group changes with fluctuations in group membership.

I wanted to know how breeder turnover, group size, and harvest affected the fluctuation of unique alleles (i.e., number of allele variants) in groups of gray wolves continuously occupying territories over time. I also wanted to know if there were differences in allelic change as a function of differences between study areas. Because wolves exhibit sex-biased male dispersal and female philopatry for inheriting breeding positions (Ausband 2022), I predicted that 1) breeding male turnover would increase the number of new unique alleles in groups and decrease the number of existing unique alleles in groups,2) breeding female turnover would have no effect on gain of new alleles but would reduce the loss of unique alleles in groups, 3) increasing group size would have no effect on unique alleles gained, but would reduce the number of unique alleles lost because at least some members that carry the existing alleles would survive from year to year in larger groups, and 4) increasing harvest (i.e., hunting and trapping) would increase both the gain and loss of unique alleles into groups due to turnover of individuals in groups. I tested these predictions in a harvested population of gray wolves in Idaho, USA using recurrent genetic sampling of wolf groups each year.

## Study area

**Fig. 1:**
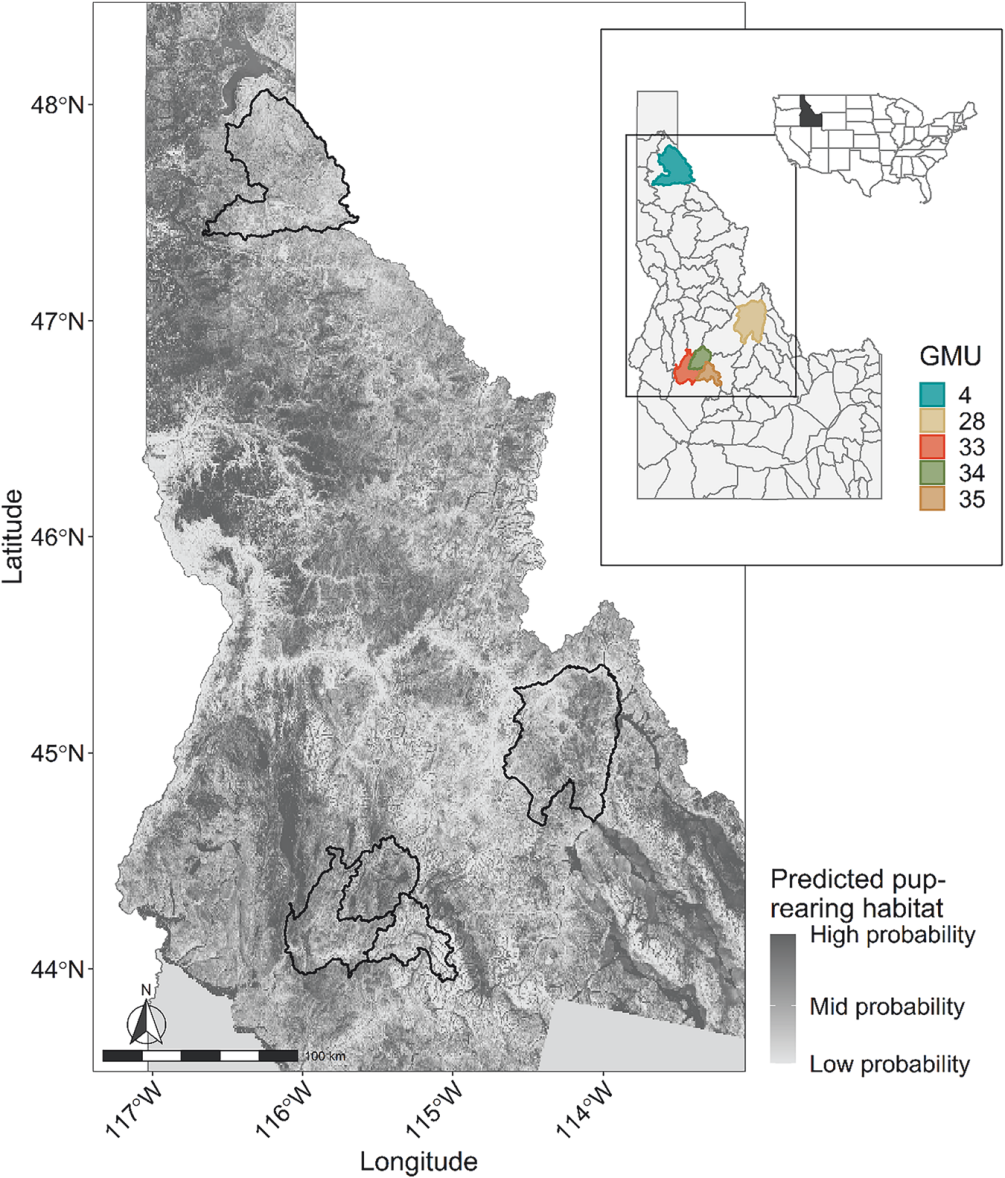
Study areas (black polygons) in Idaho, USA, where gray wolves were genetically sampled, 2008–2020. Wolves were located by annually surveying sites predicted by a pup-rearing habitat model. GMU Game Management Unit (Idaho Department of Fish and Game).

Field crews located wolf packs to obtain genetic samples in three study areas (Idaho Department of Fish and Game, Game Management Units 4, 28, and 33–35) in Idaho, USA, from 2008 to 2020 (Fig. 1). Study areas were originally chosen as part of a long-term monitoring program for recovering gray wolves in Idaho. Study areas were largely forested public land managed by the U.S. Forest Service and permanent human residences were generally few. Temperatures varied between −13 °C to 36 °C (Western Regional Climate Center 2023), precipitation varied 30–130 cm, and elevation varied 646–3219 m. The north study area (3189 km^2^, Fig. 1) was mixed conifer forests of largely western red cedar (*Thuja plicata*), Douglas fir (*Pseudotsuga menziesii*), Engelmann spruce (*Picea engelmannii*), and lodgepole pine (*Pinus contorta*). The east (3388 km^2^, Fig. 1) and south (3861 km^2^, Fig. 1) study areas were mixed forests comprising ponderosa pine (*P. ponderosa*), lodgepole pine, spruce mixed forests, and sagebrush (*Artemisia tridentata*) steppe.

### Wolf population

Overall, the wolf population in Idaho numbered roughly 1000 wolves monitored through a combination of radiocollaring and trail camera surveys. Approximately 10–14 wolf groups occupied our three study areas in any given year with 3–5 wolf groups in each study area. Hunting and trapping (wolf harvest) began in 2009 and occurred annually every year thereafter with a brief pause in 2010 due to litigation. Statewide wolf harvest has gradually been liberalized over time with an average of 309 (SD = 78) wolves removed annually by roughly equal proportions of hunting (e.g., rifle) and trapping during 2009–2020. Although poaching may occur, we do not know the rate of this potential source of mortality.

## Methods

Field staff collected wolf scats for genetic analyses at pup-rearing sites during summer from ~15 June – 30 August. Staff located wolf packs by surveying highly suitable sites predicted from a pup-rearing habitat model that reduces the overall survey area by approximately 89% (Fig. 1; Ausband et al. 2010). Briefly, at each site technicians gave a series of howls (Harrington and Mech 1982) and then searched for the activity center (i.e., area where pups congregate) where fecal samples would be most abundant. Pup scats were <2.5 cm diameter and adult scats were >2.5 cm (Weaver and Fritts 1979; Ausband et al. 2010; Stenglein et al. 2011). Staff resampled each group every year. Fieldwork was performed under University of Montana IACUC (Animal Use Protocol 008-09MMMCWRU) and University of Idaho IACUC-2018-73.

DNA analyses were conducted at the University of Idaho’s Laboratory for Ecological, Evolutionary and Conservation Genetics (Moscow, ID, USA). Mitochondrial DNA was used to remove non-target species and low-quality samples. Laboratory personnel then attempted to genotype all remaining samples using 18 nuclear DNA microsatellite loci plus a sex marker for males and females. For each locus, we required ≥2 independent polymerase chain reaction (PCR) amplifications for consensus of a heterozygote and ≥3 independent PCR amplifications for consensus of a homozygote. We compared all consensus genotypes and all unique genotypes of previously identified individuals using Program Genalex (Peakall and Smouse 2006) to match samples and distinguish unique genotypes. To avoid overestimation and account for undetected genotyping errors, we grouped samples mismatching by allelic dropout at only 1 locus (e.g., 102, 102 vs. 102, 106) as a single individual (Adams and Waits 2007). We used Program Reliotype (Miller et al. 2002) to test the accuracy of unique genotypes represented by only 1 noninvasive sample (i.e., single detections) by ensuring the genotype attained a 95% accuracy threshold. Further laboratory methods can be found in (Stenglein et al. 2010a; Stenglein et al. 2010b; Stenglein et al. 2011; Stansbury et al. 2014).

I determined parentage from pedigree analyses using Program COLONY, version 2.0.5.5 (Jones and Wang 2010). For each year, I included all adult males and females as potential parents and all pups as potential offspring. I first calculated allele frequencies for each year using Program COANCESTRY version 1.0.1.5 (Wang 2011) and then imported them into Program COLONY for use in pedigree analyses. I allowed for an allelic dropout rate of 0.01 and other genetic error rates (including mutations) of 0.01. Resulting pedigrees and recurrent annual sampling allowed tracking of individuals through time and determining if there was breeder turnover (i.e., detected at time_(t)_ but not time_(t+1)_) in groups. Lastly, I used Program Genalex 6.503 (Peakall and Smouse 2006) to calculate observed heterozygosity of individuals for each group at the start and end of their sampling timeframe (e.g., 2008 vs 2020) to see if groups became less genetically diverse over time. For each wolf group, I recorded the number of unique alleles in the group at time_(t)_ and compared it to the number of unique alleles at time_(t+1)_ then tallied the number of unique alleles gained and lost each year. I did not include pups in each year’s total because they reflected the allelic content of the breeders’ genotypes. Additionally, the number of unique alleles in a population can be affected by sample size; the more individuals sampled the more likely one observes different alleles. Although allelic richness measures can account for sample size, I wanted to track the flux of individuals alleles in and out of groups each year. Wolves live and breed in highly related family groups typically arising from a dominant breeding pair making sample size less influential on the number of unique alleles in groups. Ultimately, I modeled the gain and loss of alleles in groups and used group size in candidate models, in part, as a proxy for sample size.

I estimated annual wolf harvest density (wolves harvested/1000 km^2^) using the locations of harvested wolves in each study area to create a sum, then divided the total number harvested in each study area by study area size and finally multiplied the result by 1000 for ease of comparison to wolf density estimates commonly used in the literature (e.g., wolves/1000 km^2^).

Preliminary data exploration showed breeding male and female turnover were correlated (Pearson’s r = 0.48). Additionally, breeding female turnover and harvest were correlated (Pearson’s r = 0.35), but harvest and breeding male turnover were not (Pearson’s r = 0.17). Thus, I did not include harvest and breeding female turnover in the same model and I modeled breeding male and female turnover separately. Finally, the data were overdispersed suggesting a Poisson distribution was inappropriate. After standardizing harvest density and group size covariates using a Z-score for ease of comparison, I used a generalized linear model with a negative binomial distribution to model the number of unique alleles gained and lost between time_(t)_ and time_(t+1)_ for each wolf group as a binomial function of breeding male and female turnover between time_(t)_ and time_(t+1)_, group size at time_(t)_, and study area. I attempted to include a random effect for group using a mixed effects model approach but such models would not converge. Lastly, as a coarse measure of model fit, I modeled unique alleles gained and lost between time_(t)_ and time_(t+1)_ for each wolf group with a null model that included only an intercept. Finally, I used Akaike’s Information Criterion (AIC) to assess support between candidate models (Burnham and Anderson 2002). Analyses were conducted using the MASS package in Program R (R Core Team 2022).

## Results

I genotyped 822 adults (>1 year old) across 14 groups for a total of 106 wolf-group-years. Group size averaged 6.2 adults/group (SD = 3.1) and harvest density (wolves removed/1000 km^2^) averaged 4.1 (range: 0.0–15.4) annually. I observed 37 breeding female turnover and 40 breeding male turnover events. There were 27 instances where both the breeding female and male turned over within a group. Groups averaged 50.4 unique alleles (SD = 7.5) each year. Observed heterozygosity per group did not change over the study, 0.75 (SD = 0.06) vs. 0.71 (SD = 0.07).

**Fig. 2:**
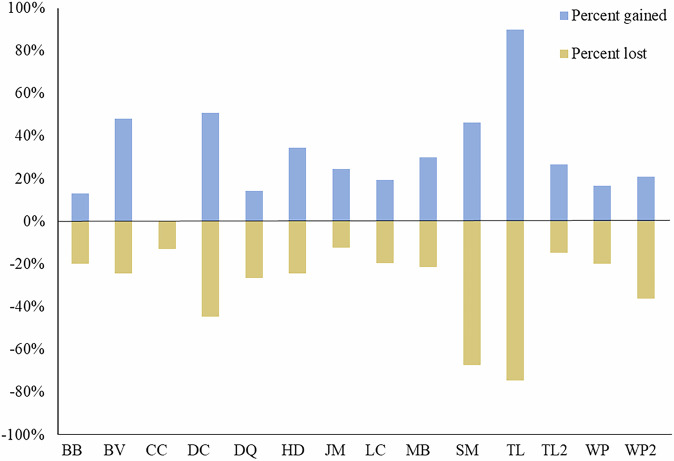
Percent of unique alleles gained (blue) and lost (tan) in gray wolf groups in Idaho, USA, 2008–2020. The x-axis labels are acronyms identifying each wolf group.

Groups gained an average of 4.3 alleles each year (SD = 6.1) totaling 8.1% (SD = 11.1) new alleles between time_(t)_ and time_(t+1)_ (Fig. 2). Groups also lost an average of 4.3 alleles each year (SD = 5.8) and retained 88.5% (SD = 19.9) of their alleles between time_(t)_ to time_(t+1)_ (Fig. 2).

**Fig. 3:**
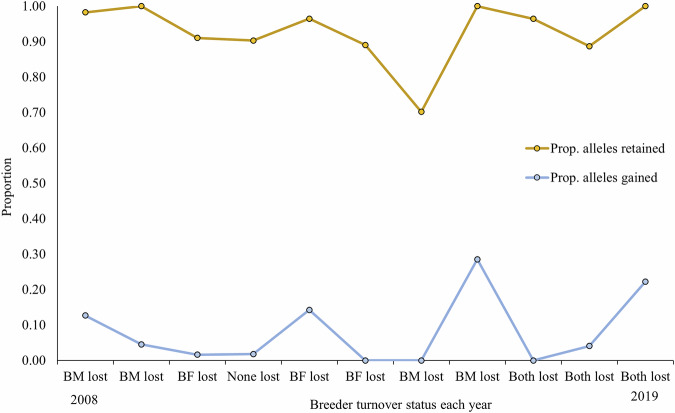
Proportion of alleles gained (blue) and retained (tan) as a function of breeder loss in an example gray wolf group (MB) in Idaho, USA, 2008–2019. BM Breeding male, BF Breeding female, Both Both sexes of breeders lost.

The top model for predicting the number of alleles gained per group each year included breeding male turnover, although a candidate model that included breeding male turnover and harvest was equally supported (within 2.0 AICs). Harvest was not a significant predictor of alleles gained in this model (Tables 1 and 2). The top model for predicting the number of alleles lost per group each year included breeding male turnover, although two candidate models that included harvest and group size were equally supported (Tables 3 and 4; Fig. 3). Both harvest and group size were not significant predictors of allelic change in these models.

**Table 1:** Candidate models for predicting the number of unique alleles gained in groups of gray wolves in Idaho, USA, 2008–2020.

	Model	*K*	*−2LL*	AIC	ΔAIC	AIC*w*_*i*_
1.	Breeding male turnover	3	421.6	427.6	0	0.60
2.	Breeding male turnover + Harvest density	4	421.2	429.2	1.6	0.27
3.	Breeding male turnover + Group size + Harvest density	5	421.1	431.1	3.5	0.10
4.	Breeding male turnover + Group size + Harvest density + Study area	6	420.8	434.8	7.2	0.02
5.	Breeding female turnover	3	431.9	437.9	10.3	0.0
6.	Breeding female turnover + Group size	4	431.8	439.8	12.2	0.0
7.	Null	2	437.8	441.8	14.2	0.0
8.	Breeding female turnover + Group size + Study area	5	430.9	442.9	15.3	0.0

**Table 2:** Covariates from equally supported top models (i.e., within 2.0 AICs) for predicting the number of unique alleles gained in groups of gray wolves in Idaho, USA, 2008–2020.

	ΔAIC	Covariate	*β*	SE	*P*
Model 1	0.0	Intercept	0.64	0.22	0.003
		Breeding male turnover	1.35	0.31	<0.0001
Model 2	1.6	Intercept	0.64	0.22	0.003
		Breeding male turnover	1.33	0.32	<0.0001
		Harvest density	0.09	0.16	0.58

**Table 3:** Candidate models for predicting the number of unique alleles lost in groups of gray wolves in Idaho, USA, 2008–2020.

	Model	*K*	*−2LL*	AIC	ΔAIC	AIC*w*_*i*_
1.	Breeding male turnover	3	427.9	433.9	0	0.47
2.	Breeding male turnover + Harvest density	4	427.7	435.7	1.8	0.19
3.	Breeding male turnover + Group size + Harvest density	5	425.8	435.8	1.9	0.18
4.	Breeding female turnover	3	442.0	438.0	4.1	0.06
5.	Breeding male turnover + Group size + Harvest density + Study area	7	424.1	438.1	4.2	0.06
6.	Breeding female turnover + Group size	4	431.9	439.9	6.0	0.02
7.	Breeding female turnover + Group size + Study area	6	430.2	442.2	8.3	0.01
8.	Null	2	440.8	444.8	10.9	0.0

**Table 4:** Covariates from top equally supported models (i.e., within 2.0 AICs) for predicting the number of unique alleles lost in groups of gray wolves in Idaho, USA, 2008–2020.

	ΔAIC	Covariate	*β*	SE	*P*
Model 1	0.0	Intercept	0.76	0.22	0.0001
		Breeding male turnover	1.19	0.31	0.0002
Model 2	1.8	Intercept	0.77	0.22	0.004
		Breeding male turnover	1.16	0.32	<0.0001
		Harvest density	0.06	0.16	0.68
Model 3	1.9	Intercept	0.67	0.22	0.002
		Breeding male turnover	1.32	0.32	<0.0001
		Group size	0.23	0.16	0.16
		Harvest density	0.11	0.16	0.51

## Discussion

Breeding opportunities can be rare in populations of animals that live and breed in groups (Hatchwell and Komdeur 2000). Thus, we might expect relatively stable allelic compositions in family groups of such species. I show that over time groups often gain and lose alleles in roughly equal proportions, but there are episodic changes to alleles in groups particularly as a function of breeding male turnover.

The most consistent driver of allelic change in groups was breeding male turnover. As expected, breeding male turnover had a strong positive effect on the number of alleles gained per group, far stronger than other variables I considered. Wolves exhibit sex-biased dispersal and these findings support the idea that males are largely the genetic couriers in established wolf populations (VonHoldt et al. 2008; Wikenros et al. 2021; Ausband 2022). Contrary to my predictions, breeding female turnover had significant positive effects on both alleles gained and lost in groups, although models with breeding female turnover had far less support than models with breeding male turnover. Because female wolves are philopatric and largely queue for breeding opportunities (Sillero-Zubiri et al. 1996; Ausband 2022; Pacheco et al. 2024), I posited they would help retain their parents’ allelic lineage and, on average, not foster new alleles into groups. Twenty-seven breeding female turnover events also included breeding male turnover within the group, however. Thus, the allelic fluctuations observed after breeding female turnover may have been affected by concurrent breeding male turnover in some years.

Harvest density ranged widely over study areas and years and included years with no harvest and others where harvest was as high as 15.4 wolves/1000 km^2^. Indeed, such harvest rates often exceed wolf density estimates in the literature (Fuller et al. 2003). We would expect large demographic and genetic effects from such high harvest rates. Perhaps surprising then, although harvest was correlated with breeding female turnover, it had no effect on allelic change in groups. Harvest was commonly in equally supported models as an insignificant covariate. While I predicted that harvest would affect alleles lost, I also predicted it would affect alleles gained by creating turnover with groups and potentially increasing immigration and the arrival of new alleles into groups (e.g., Chen et al. 2019). My prediction, arising from group augmentation theory (Kokko et al. 2001), assumed that individuals in groups would be more likely to accept adoptees into their group as harvest increased. Groups did not readily accept adoptees, however, and only did so when there was a breeding vacancy. Occasionally, the arrival of an adoptee at time_(t)_ preceded breeder turnover with the adoptee then becoming a breeder at time_(t+1)_. Nonbreeding adoptees were exceedingly rare (*n* = 5) and some groups (*n* = 8) disbanded after reaching just a few animals. Most of these territories remained vacant for several years until new, unrelated wolves reoccupied them (5 of the 8 territories). This finding supports previous work on wolves that showed immigration had a strong influence on the growth and persistence of harvested population (Adams et al. 2008). Although my fine-scale assessment at the group level showed periodic evidence of group extinction and vacant territories, after 1–3 years, new wolves usually (but not always) immigrated and reestablished a breeding group. Such a finding likely applies to large, contiguous populations of wolves such as those currently found in the Rocky Mountains of the U.S. (U.S. Fish and Wildlife Service 2023).

I predicted increasing group size would mitigate the loss of alleles in groups, but that did not occur. I note, however, that I did not include pups in annual allele tallies, and they likely would have bolstered the number of alleles retained each year assuming at least some pups survive to one year old. Additionally, the allelic composition of groups can change as individuals arrive and leave groups. But they can also change because of random mutations or genotyping errors (Allendorf et al. 2022). While my parentage analyses accounted for allelic dropout and random mutations, I cannot rule out that in some years small amounts of the allelic change I observed were due to such factors.

I found considerable annual allelic variation in groups of cooperative breeders. Much of this variation was attributable to breeding male turnover. While groups generally gained and lost alleles in equal proportions over time, such gains and losses were episodic within years. These findings have implications for how we define and evaluate group persistence, family groups, and breeder lineages in cooperative breeders. For example, group “TL” lost nearly 80% of its alleles between time_(t)_ and the end of the monitoring (Fig. 2); conversely, in other cases substantial numbers of the group’s original alleles may be maintained long after all original group members have left (due to mortality or otherwise). In either case, it is unclear if the group “identity” (i.e., is it the “same” group) is maintained. Defining group identity consistently has important implications for studying the evolution and maintenance of cooperative breeding. Although I do not answer these questions here, it may be beneficial to define characteristics and vital rates of groups based, at least in part, on their underlying genetics when such information can be obtained.

## Supplementary information

R script for analyses

## Data Availability

Data are freely available at Ausband, D. (2025). Ausband allele data, gray wolves [Data set]. Zenodo. 10.5281/zenodo.16328526.
